# Novel therapeutic strategies targeting myeloid-derived suppressor cell immunosuppressive mechanisms for cancer treatment

**DOI:** 10.37349/etat.2024.00212

**Published:** 2024-02-28

**Authors:** Eric Jou, Natasha Chaudhury, Fizza Nasim

**Affiliations:** Kumamoto University, Japan; ^1^Medical Sciences Division, Oxford University Hospitals, University of Oxford, OX3 9DU Oxford, UK; ^2^Kellogg College, University of Oxford, OX2 6PN Oxford, UK; ^3^Wexham Park Hospital, Frimley Health NHS Foundation Trust, SL2 4HL Slough, UK

**Keywords:** Tumor microenvironment, cancer therapy, preclinical models, immunotherapy, myeloid-derived suppressor cell, myeloid cells, immunosuppression

## Abstract

Cancer is the leading cause of death globally superseded only by cardiovascular diseases, and novel strategies to overcome therapeutic resistance against existing cancer treatments are urgently required. Myeloid-derived suppressor cells (MDSCs) are immature myeloid cells with potent immunosuppressive capacity against well-established anti-tumour effectors such as natural killer cells (NK cells) and T cells thereby promoting cancer initiation and progression. Critically, MDSCs are readily identified in almost all tumour types and human cancer patients, and numerous studies in the past decade have recognised their role in contributing to therapeutic resistance against all four pillars of modern cancer treatment, namely surgery, chemotherapy, radiotherapy and immunotherapy. MDSCs suppress anti-tumour immunity through a plethora of mechanisms including the well-characterised arginase 1 (Arg1), inducible nitric oxide synthase (iNOS) and reactive oxygen species (ROS)-mediated pathways, along with several other more recently discovered. MDSCs are largely absent in healthy homeostatic states and predominantly exist in pathological conditions, making them attractive therapeutic targets. However, the lack of specific markers identified for MDSCs to date greatly hindered therapeutic development, and currently there are no clinically approved drugs that specifically target MDSCs. Methods to deplete MDSCs clinically and inhibit their immunosuppressive function will be crucial in advancing cancer treatment and to overcome treatment resistance. This review provides a detailed overview of the current understandings behind the mechanisms of MDSC-mediated suppression of anti-tumour immunity, and discusses potential strategies to target MDSC immunosuppressive mechanisms to overcome therapeutic resistance.

## Introduction

Cancer is the second leading cause of death globally despite contemporary advances in medicine. Immunotherapies have completely revolutionised cancer treatment in the past decade showing unprecedented efficacy against many tumour types, however, not all patients respond [1]. Overcoming immunotherapy resistance has emerged as one of the most crucial questions in medicine in the current era.

While the immune system plays critical roles in protecting the human body from cancer in a process known as cancer immunosurveillance, established tumours that present clinically harbour an immunosuppressive tumour microenvironment which prevents anti-tumoural immunity and allow disease progression and metastasis [2]. Myeloid-derived suppressor cells (MDSCs) are immature myeloid cells with potent immunosuppressive properties, and are predominantly found under disease settings such as in cancer where they are recruited to suppress anti-tumour immunity through a myriad of mechanisms [3, 4]. Evolutionarily, MDSCs have also been found transiently in the first few weeks of life in human neonates in a lactoferrin-dependant manner, which is distinct to their mechanism of accumulation in cancer, and play critical roles in protecting newborns from excessive inflammation during gut microbiota colonisation [4]. MDSCs can be broadly divided into monocytic MDSCs (M-MDSCs) or granulocytic MDSCs (G-MDSCs) based on their resemblance to monocytes and neutrophils respectively, and have been shown to suppress all components of the anti-tumour type 1 immune response including cytotoxic CD8^+^ T cells, T helper 1 (Th1) CD4^+^ T cells, NK cells, and type 1 innate lymphoid cells (ILC1s) [5, 6]. Mechanistically, MDSC-mediated inhibition of anti-tumour immunity occurs through a myriad of pathways including expression of arginase 1 (Arg1), inducible nitric oxide synthase (iNOS) and reactive oxygen species (ROS), and secretion of immunosuppressive cytokines such as interleukin 10 (IL-10). MDSCs are identified in almost all cancer patients, and increased MDSCs are uniformly associated with poor prognosis across different cancer types [7]. Critically, MDSCs have been shown to contribute to therapeutic resistance against all forms of cancer therapies, including chemotherapy, radiotherapy and immunotherapy, across multiple cancer types [8–11]. Importantly, due to the lack of specific markers, there are currently no clinically approved drugs that specifically deplete MDSCs, and the development of targeted therapy against MDSCs have proved challenging. Nevertheless, strategies that target many of the aforementioned immunosuppressive pathways utilised by MDSCs are actively being developed.

Recent animal studies have significantly advanced the fundamental understanding on how MDSCs suppress anti-tumour immunity and identifying new avenues for potential therapeutic targeting. While human studies continue to show MDSCs to have important prognostic value in predicting poor outcome and response to the therapy. This literature review provides a comprehensive discussion of the different subtypes of MDSCs, the recent advances in the understandings of MDSC immunosuppressive mechanisms, and the exciting prospect of targeting them therapeutically using a combination of strategies to overcome cancer therapeutic resistance.

## Functional overview of MDSCs in cancer

MDSCs are pathological, immature myeloid cells with potent pro-tumorigenic properties and can be readily identified in human cancer patients and tumour-bearing mice [3]. In cancer mouse models, MDSCs are classified into two main subtypes including CD11b^+^Ly6C^high^Ly6G^–^ M-MDSCs and CD11b^+^Ly6C^low^Ly6G^+^ G-MDSCs, which show resemblance in terms of surface marker expression to monocytes and neutrophils respectively [12]. In human cancer patients, similar subsets of MDSCs exist and are characterised by different surface markers along with reduced human leukocyte antigen-DR isotype (HLA-DR) expression, namely CD3^–^CD19^–^CD56^–^CD11b^+^CD14^+^CD15^–^CD33^+^HLA-DR^low/–^ M-MDSCs, and CD3^–^CD19^–^CD56^–^CD11b^+^ CD14^–^CD15^+^CD33^+^HLA-DR^low/–^ G-MDSCs. As alluded to, MDSCs have been identified in almost all types of human cancers associating with poor prognosis [7], and can be found both in the peripheral blood of cancer patients serving as a relatively “easy-to-access” prognostic biomarker as well as within the tumour microenvironment.

**Figure 1:**
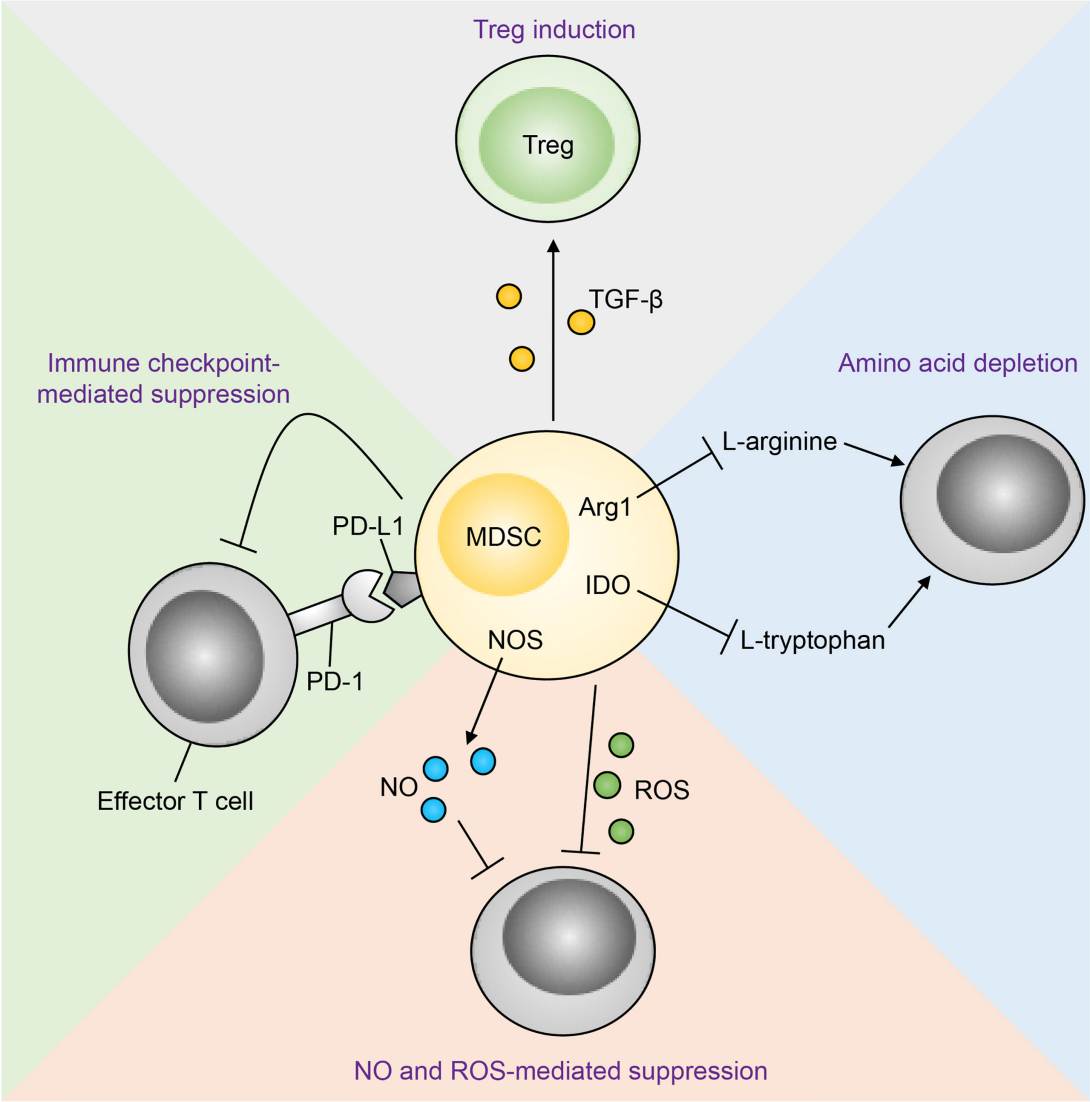
Mechanisms of MDSC-mediated immunosuppression of anti-tumour immunity [112]. Treg: regulatory T cell; TGF-β: transforming growth factor β; PD-1: programmed cell death protein 1; NOS: nitric oxide synthase; NO: nitric oxide; IDO: indoleamine 2,3-dioxygenase *Note.* Reprinted from “Emerging roles for IL-25 and IL-33 in colorectal cancer tumorigenesis,” by Jou E, Rodriguez-Rodriguez N, McKenzie ANJ. Front Immunol. 2022;13:981479 (https://www.frontiersin.org/articles/10.3389/fimmu.2022.981479/full). CC BY.

MDSCs have been shown to suppress anti-tumour immunity through a plethora of mechanisms as highlighted by mechanistic studies utilising animal cancer models and cells from human cancer patients *ex vivo* (Figure 1). These include high expression of Arg1, iNOS and ROS, downregulation of major histocompatibility complex (MHC) class II (MHC-II), production of immunosuppressive cytokines, and expression of a myriad of inhibitory immune checkpoint ligands such as CD80, CD86, programmed cell death ligand 1 (PD-L1), herpesvirus entry mediator (HVEM), CD112 and CD155 [13–15]. Furthermore, studies have shown that M-MDSCs and G-MDSCs preferentially utilise different immunosuppressive mechanisms from the aforementioned list, and as such their functions are non-redundant and show potentiation through inhibiting distinct anti-tumoural pathways [16].

## Arg1-mediated MDSC immunosuppression

M-MDSCs and G-MDSCs differ from monocytes and neutrophils both phenotypically and functionally as detailed in a recent review [4], and their ability to suppress anti-tumour immunity is reflected by their distinct transcription and proteomic profiles favouring the expression of immunosuppressive genes [16]. One of the best characterised immunosuppressive mechanisms utilised by MDSCs is the high expression of Arg1, the latter being a defining feature of MDSCs phenotypically [12]. Arg1 converts the amino acid arginine into proline and the polyamine spermine, which in turn promotes tissue repair and induces cell proliferation respectively [17, 18]. Mechanistically, polyamines enhance cell proliferation through inducing cell cycle progression, providing protection against DNA damage, and preventing apoptosis [19–21]. In the context of cancer, however, they instead directly promote neoplastic cell replication resulting in tumour growth and progression [22]. Similarly, the tissue repair effects of proline are pro-tumorigenic, and accordingly elevated proline biosynthesis gene expression has been associated with poor prognosis in cancer patients [23]. Cancer cells can utilise proline as an energy source and as a precursor for protein synthesis, in particular collagen. Collagen binds to an array of receptors such as integrin and discoidin domain receptors resulting in the activation of oncogenic mitogen-activated protein kinase (MAPK) and phosphoinositide 3-kinase (PI3K) pathways, along with inducing epithelial-mesenchymal transition further promoting tumour growth and dissemination. Finally, arginine is a critical substrate required for optimal T cell and NK cell anti-tumour effector functions [24]. Experimentally, human NK cell cytotoxic activity *ex vivo* was significantly inhibited in an environment with low arginine concentration compared to when arginine is abundant, and showed reduced expression of activating receptors and impaired interferon γ (IFNγ) production [25]. Furthermore, arginine modulates T cell metabolism through favouring oxidative phosphorylation over glycolysis which in turn enhances T cell survival and facilitates the development of long-lasting memory T cells with heightened anti-tumoural properties. Depletion of arginine by MDSC Arg1, therefore, indirectly inhibits T cell and NK cell intratumoural number and function thereby suppressing anti-tumour immunity [24, 26].

Arg1 expression in MDSCs is induced by the type 2 immune cytokines IL-4 and IL-13 through signal transducer and activator of transcription 6 (STAT6)-mediated signalling [27–29]. A recent study revealed ILC2s to be the major source of IL-4 and IL-13 in tumours in a mouse model of colorectal cancer (CRC), and that ILC2-derived IL-4 and IL-13 promote M-MDSC expression both *in vitro* and *in vivo* [28]. In that study, paired comparison of tumour and spleen M-MDSCs isolated from CRC mice demonstrated that tumour M-MDSCs expressed higher levels of Arg1 than spleen M-MDSCs. Accordingly, other studies found tumour MDSCs to have superior immunosuppressive functions over MDSCs found in the periphery in line with the heightened Arg1 expression [3], and indeed tumour MDSCs strongly express the IL-4 and IL-13 receptors [28, 30]. Importantly, *in vitro* antibody-mediated neutralisation of ILC2-derived IL-4 and IL-13 reduced tumour MDSC Arg1 levels, and similarly genetic deletion of IL-13 in mice with CRC decreased Arg1^+^ MDSCs, increased IFNγ production by Th1 cells and CD8^+^ T cells, and significantly reduced tumour burden [28]. In the same study, analysis of human CRC samples revealed M-MDSCs to positively correlate with ILC2 abundance in tumours, while associating with reduced anti-tumoural T cell infiltration. Bladder cancer patients with a T cell to MDSC ratio of less than 1 have poor prognosis, and are associated with the presence of ILC2s and detectable IL-13 [30]. Collectively, these studies demonstrate that the ILC2-derived type 2 cytokines play a key role in enhancing MDSC-mediated T cell suppression through increasing Arg1 expression.

Other pathways involved in MDSC Arg1 expression include the microRNA-107 (miR-107) pathway which induces expansion and Arg1 levels of MDSCs in gastric cancer [31]. Mechanistically, gastric cancer-derived exosomes contain miR-107, which can then act on MDSCs directly thereby creating a immunosuppressive tumour microenvironment. Other miRNAs including miR-30a expression has also been shown to be associated with heightened suppressive capacities of both M-MDSCs and G-MDSCs in mouse models of B cell lymphoma via degrading suppressor of cytokine signalling 3 (SOCS3) thereby enhancing STAT3 expression and downstream Arg1 [32].

In humans, MDSC frequency is increased in the peripheral blood of patients with oesophageal cancer compared to healthy controls (15.21% and 1.10%, respectively, *P* < 0.0001), along with plasma Arg1 level (28.28 ng/mL and 9.57 ng/mL, respectively, *P* = 0.0003) [33]. Arg1 level positively correlated with serum IL-6 (*r* = 0.6404, *P* = 0.0031), consistent with the ability of IL-6 to induce Arg1 expression in MDSCs via a STAT3-mediated pathway [34]. In patients with diffuse large B-cell-lymphoma, high expression of triggering receptor expressed on myeloid cells 2 (TREM2) on circulating MDSCs is associated with Arg1 expression, enhanced immunosuppressive activity against CD8^+^ T cells and poor prognosis including reduced progression-free survival (PFS) and overall survival (OS) [35].

### Therapeutic targeting of the MDSC-Arg1 immunosuppressive pathway

**Figure 2:**
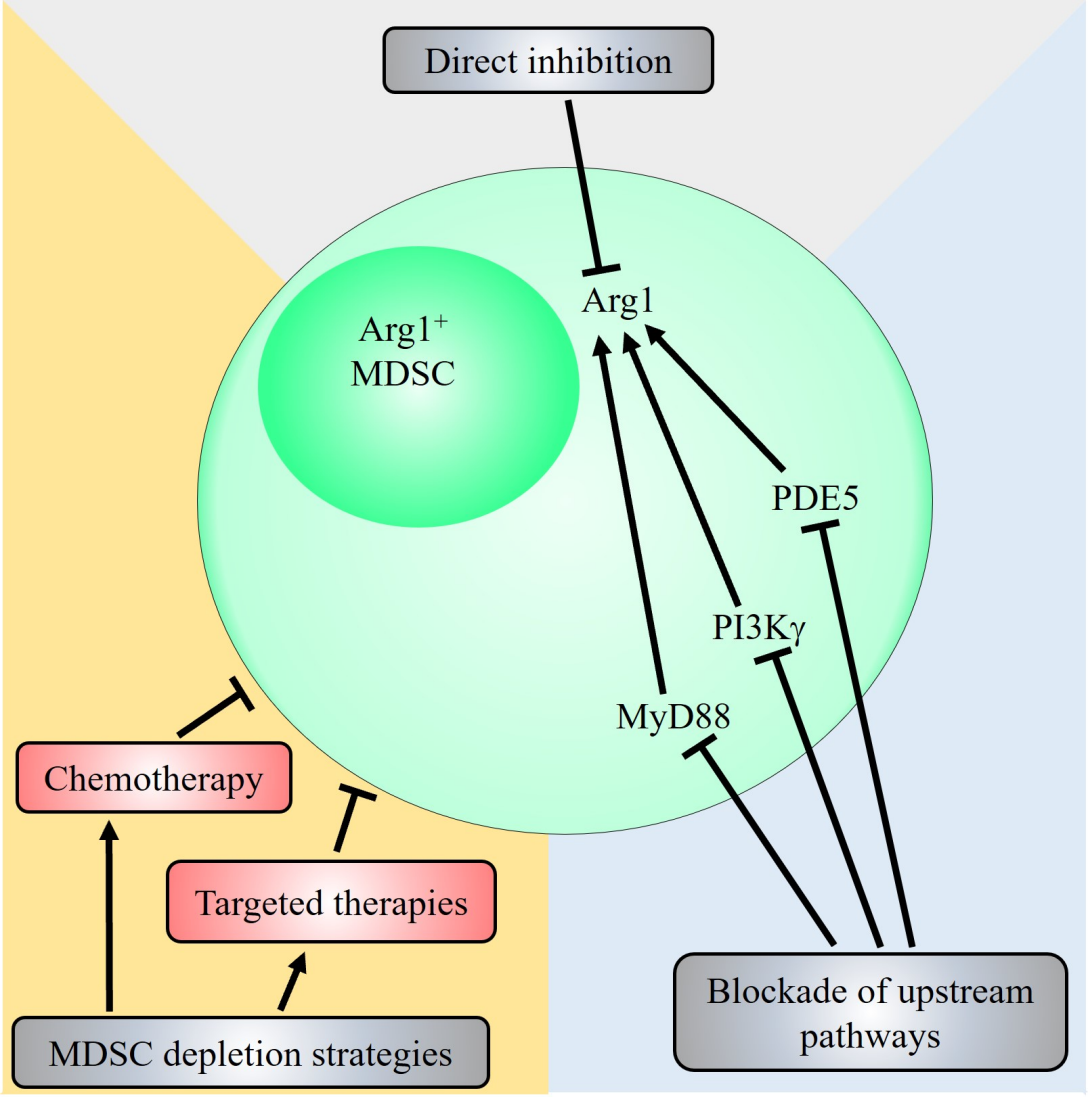
Potential therapeutic strategies targeting the MDSC-Arg1 immunosuppressive pathway. Strategies that target Arg1^+^ MDSCs include direct inhibition of Arg1 via Arg inhibitors such as *N*-hydroxy-nor-L-Arg (nor-NOHA) and CB-1158, or through blockade of upstream signalling pathways that induce Arg1 expression. Pharmacological inhibition of PDE5, PI3Kγ and MyD88 have all been shown to decrease Arg1 expression and reduce immunosuppressive activity of MDSCs. Other general strategies that directly deplete Arg1^+^ MDSCs have also been demonstrated. The chemotherapeutic agents doxorubicin or 5-FU, and targeted therapies against receptor tyrosine kinases such as anlotinib, cabozantinib and selumetinib have been shown to reduce Arg1^+^ MDSCs contributing to their anti-tumoural therapeutic efficacy

Arg1 plays a critical, non-redundant role in mediating MDSC immunosuppressive function as highlighted by many studies, and is an attractive therapeutic target (Table 1). While M-MDSCs express high levels of NO and low levels of ROS and G-MDSCs show an opposite profile instead preferentially express ROS over NO, both subsets of MDSCs express high levels of Arg1 [12]. In light of this, many potential therapeutic strategies to inhibit Arg1 or reduce Arg1^+^ MDSCs have been proposed (Figure 2).

**Table 1:** Studies and strategies targeting the MDSC-Arg1 pathway

**Study**	**Cancer type**	**Strategy to suppress MDSC function**
Ding et al. [39]	Colon cancer	IPI-549 inhibits PI3Kγ in MDSCs leading to downregulation of Arg1 and ROS, and promote MDSC apoptosis. The resultant activation of CD8^+^ T cells reduced tumour recurrence and metastasis.
Yu et al. [113]	Hepatocellular carcinoma (HCC)	Treatment with PDE5 inhibitor suppressed MDSC immunosuppressive functions via inhibiting Arg1 and iNOS in HCC models.
Yuan et al. [47]	Lung cancer	Addition of anlotinib to radiotherapy and anti-PD-1 treatment reduced MDSCs and Arg expression leading to enhanced CD8^+^ T cell infiltration and IFNγ production.
Wang et al. [44]	Multiple cancer types	P300-mediated C/EBPβ acetylation enhances MDSC Arg1 expression and immunosuppressive function. Pharmacological inhibition of P300 may be of benefit to target MDSCs although this was not formally tested in the study.
Wang et al. [35]	Diffuse large B-cell lymphoma (DLCBL)	In treatment-naive DLBCL adults, a high M-MDSC surface expression of TREM2 is a poor prognostic factor for both PFS and OS, highlighting TREM2 as a potential therapeutic target.
Khaki Bakhtiarvand et al. [48]	Breast cancer	Addition of cabozantinib to anti-HER2 treatment enhanced IFNγ levels and reduced tumour growth via inhibition of Arg1^+^ MDSCs.
Li et al. [41]	Lung cancer	Sanguinarine (SNG) treatment inhibits MDSC suppressive function via reducing Arg1, iNOS and ROS expression.
Zamani et al. [45]	Breast cancer	Doxorubicin chemotherapy reduced MDSC frequency, Arg1, iNOS and ROS expression, and enhanced anti-tumour T cell tumour infiltration.
Steggerda et al. [38]	Multiple cancer types	Arg inhibitor CB-1158 treatment inhibited MDSC-mediated T cell suppression leading to reduced tumour growth in multiple murine cancer models, either alone or in combination with immunotherapies such as immune checkpoint inhibitors, adoptive T cell or NK cell transfer and chemotherapy.
Wang et al. [42]	CRC (colitis-associated)	Blocking MyD88 with the novel inhibitor TJ-M2010-5 suppressed MDSC Arg1 and iNOS expression enhancing anti-tumour immunity.
Otvos et al. [46]	Gliobastoma	Depletion of Arg1^+^ MDSCs via blocking migration inhibitory factor (MIF) or through a low-dose 5-flurouracil (5-FU) regime resulted in prolonged survival in a mouse model of glioma.
Le Noci et al. [114]	Melanoma lung metastases	Nebulised anti-MDSC antibody RB6-8C5 treatment reduced mRNA levels of Arg1, leading to NK cell activation and enhanced tumour reduction.
Poon et al. [49]	CRC	Anti-CTLA-4 immune checkpoint inhibition increased Arg1 expression in the tumour microenvironment. The increase in Arg1 expression can be effectively reversed by combination treatment with selumetinib, the latter suppressing MDSCs, leading to potentiation and enhanced anti-tumour efficacy of immune checkpoint blockade.
Thakur et al. [50]	Pancreatic cancer	Combination treatment using anti-CD3 and anti-HER2 bispecific antibody together with EGFR inhibitor reduced MDSC Arg1 expression, leading to increased levels of Th1 cytokine-mediated anti-tumour immunity.

PDE5: phosphodiesterase 5; C/EBPβ: CCAAT/enhancer-binding protein β; HER2: human epidermal growth factor receptor 2; MyD88: myeloid differentiation factor 88; mRNA: messenger RNA; CTLA-4: cytotoxic T-lymphocyte antigen-4; EGFR: epidermal growth factor receptor

Arg1 function can be directly inhibited by Arg1 inhibitors. Treatment with the Arg1 inhibitor nor-NOHA abrogated MDSC-mediated T cell suppression experimentally [34, 36], and restored the proliferation of MDSC-suppressed lymphocytes from patients with multiple myeloma or head and neck squamous cell carcinoma (HNSCC) [37]. Inhibition of Arg1 via CB-1158 prevented MDSC-mediated suppression of T cell proliferation *in vitro*, and reduced tumour growth in multiple murine models of cancer via potentiating the effects of the chemotherapy gemcitabine and immunotherapies including immune checkpoint inhibitors, adoptive T cell or NK cell transfer [38].

Other strategies include targeting upstream pathways responsible for MDSC Arg1 expression thereby reducing the numbers of Arg1^+^ MDSCs. In CRC, inhibition of PI3Kγ in MDSCs downregulates Arg1 and ROS leading to MDSC apoptosis and reduction in immunosuppression capacity, thereby allowing CD8^+^ T cells activation and tumour elimination [39]. In HCC studies, MDSCs suppressed cytotoxic activity of cell-based immunotherapy cytokine-induced killer (CIK), and concomitant treatment with a PDE5 inhibitor reduced tumour MDSCs numbers and suppressor function by downregulating Arg1 and iNOS potentiating tumour eradication both *in vitro* and *in vivo* [40]. SNG treatment suppressed MDSCs *in vitro* inducing apoptosis and downregulation of Arg1, iNOS and ROS in a Lewis lung cancer mouse model, and promoted MDSC differentiation into mature macrophages [41]. This resulted in a significant increase in anti-tumoural T cells including Th1 cells and cytotoxic T lymphocytes, and a reduction in tumour burden. Others have found that MyD88 inhibition via a novel inhibitor TJ-M2010-5 prevented CRC in a mouse model of colitis-associated cancer (CAC) via suppression of MDSC Arg1, iNOS and IDO expression [42]. In that study, using the azoxymethane/dextran sodium sulphate (AOM/DSS) model of CAC which mirrors inflammatory bowel disease-associated CRC in humans, MDSCs were found to be expanded in tumour-bearing mice in a MyD88-dependent manner. Inhibition of MyD88 led to significant reductions in key cytokines associated with MDSC expansion including granulocyte-colony stimulating factor (G-CSF), IL-6, IL-1β and TGF-β, leading to fewer MDSCs and reduced cancer burden. Furthermore, cholesterol deficiency was found to enhance the immunosuppressive capacity of MDSCs [43]. Mechanistically, using a receptor-interacting protein kinase 3 (RIPK3)-deficient MDSC model, the authors showed that reduced cholesterol in tumour-infiltrating MDSCs led to increased nuclear liver X receptor beta (LXRβ), which in turn forms a heterodimer with retinoid X receptor α (RXRα) thereby directly inducing Arg1 expression via LXRβ-RXRα binding to the Arg1 promoter. Accordingly, inhibition of cholesterol synthesis via itraconazole-mediated suppression of the upstream RIPK3-protein kinase B (AKT)-mammalian target of rapamycin complex 1 (mTORC1) pathway enhanced MDSC immunosuppressive activity and increased tumour burden. Therefore, strategies that modify cholesterol synthesis may allow modulation of MDSC function. Additionally, the histone lysine acetyltransferase p300 transcription factor has been found to induce Arg1 expression at the transcription level via C/EBPβ acetylation, thereby enhancing MDSC suppressive capacity, and may be a potential future therapeutic target [44]. Mechanistically, p300-mediated acetylation of C/EBPβ leads to enhanced C/EBPβ transactivation activity on the Arg1 promoter subsequently enhancing Arg1 expression, and pharmacological inhibition of p300 led to reduced MDSC immunosuppressive activity and tumour burden *in vivo*.

Arg1^+^ MDSCs can also be targeted via chemotherapy, one of the four pillars of modern cancer treatment. Doxorubicin is routinely used for breast cancer treatment in patients as a chemotherapeutic agent, and mechanistically reduces expression of MDSC-associated genes including Arg1, iNOS, S100A8 and S100A9 in breast tumour mouse models [45]. S100A8 and S100A9, like Arg1, are associated with heightened MDSC immunosuppressive capacity [28]. Depletion of MDSCs via low-dose 5-FU treatment increased the survival of mice with glioma driven by Arg1^+^ MDSCs [46]. Similarly, MDSCs are associated with reduced immunotherapy efficacy, and combination treatment targeting MDSCs may overcome therapeutic resistance. In a mouse model of lung cancer, triple therapy via addition of the targeted therapy anlotinib [which inhibits the vascular endothelial growth factor receptor (VEGFR)] to radiotherapy and immunotherapy (anti-PD-L1 immune checkpoint inhibition), reduced MDSCs and Arg1 levels resulting in significantly more CD8^+^ T cell infiltration, heightened IFNγ expression, and lower tumour burden compared to without anlotinib [47]. Accordingly, addition of anlotinib similarly led to a reduction in expression of MDSC-associated immunosuppressive cytokines including IL-10 suggesting impaired MDSC function. The targeted therapy drug cabozantinib which inhibits multiple receptor tyrosine kinases has been shown to reduce MDSC Arg1 expression and frequency by 20%, 0.8% and 35% in the tumour microenvironment, lymph nodes and spleen respectively in breast cancer mice [48]. Furthermore, combination treatment with anti-HER2 showed potentiation resulting in heightened tumour eradication consistent with the ability of MDSCs to contribute to treatment resistance against existing gold-standard therapies. In the CT26 mouse model of CRC, immune checkpoint inhibition via anti-CTLA-4 increased Arg1 expression in the tumour microenvironment. The increase in Arg1 expression is effectively negated through the addition of the mitogen-activated protein kinase kinase (MEK) inhibitor selumetinib, which acts via reducing Arg1-expressing MDSCs resulting in enhanced treatment efficacy [49]. Similarly, treatment with EGFR inhibitor targeted therapy suppresses MDSCs and Arg1 expression, resulting in heightened levels of Th1 cytokines such as IL-12 and tumor necrosis factor-alpha (TNF-α) and reduced pancreatic cancer [50]. Altogether, these studies indicate that therapeutic targeting of Arg1^+^ MDSCs, either alone or in combination with existing therapies, can effectively reduce cancer burden and warrants further exploration.

## iNOS in MDSC-mediated immunosuppression and potential therapeutic strategies

Of the three isoforms of NOS, iNOS is unique in that it is not constitutively expressed and instead induced in response to inflammatory stimuli, such as in autoimmune disease and cancer. Studies in CRC patients found plasma iNOS concentration to positively correlate with MDSC frequency and have been proposed to act as a surrogate for measuring MDSC abundance [51]. In a systematic review and meta-analysis of 14 studies encompassing a total of 1,758 patients with solid tumours, increased iNOS expression was found to be associated with significantly worse OS [hazard ratio (HR) = 1.89; 95% confidence interval (CI): 1.57–2.28; *P* ≤ 0.001], cancer-specific survival (HR = 3.13; 95% CI: 1.88–5.20; *P* ≤ 0.001) and recurrence free survival (HR = 2.16; 95% CI: 1.29–3.62; *P* = 0.003). Indeed, many studies have found iNOS to play critical roles in mediating MDSC immunosuppressive function. Unlike Arg1 expression driven by type 2 cytokines, iNOS expression in MDSCs is induced in response to the type 1 immune cytokine IFNγ [52], the latter classically being ascribed an anti-tumoural role. In fact, whilst IFNγ as part of the type 1 immune response has well-established anti-tumoural functions, recent studies have indicated that in certain chronic inflammatory contexts IFNγ may have pro-tumoural functions [53, 54]. A recent study showed that hyperactivation of type 1 immunity can instead lead to overexpression of iNOS in MDSCs and counterproductively induces strong negative feedback to suppress anti-tumour type 1 immunity [55]. It is, however, worth noting that in most cancer types, the anti-tumoural activities of IFNγ largely outweighs it’s pro-tumoural effects, and indeed increased IFNγ expression is amongst the strongest positive prognostic indicator of improved patient survival across a broad range of cancer types [56–62]. This, therefore, raises the exciting prospect of combining iNOS inhibitors (or other strategies that target iNOS^+^ MDSCs) with IFNγ-based cancer therapies, which may show potentiation by removing the pro-tumoural aspect of IFNγ while preserving the anti-tumoural functions.

**Figure 3:**
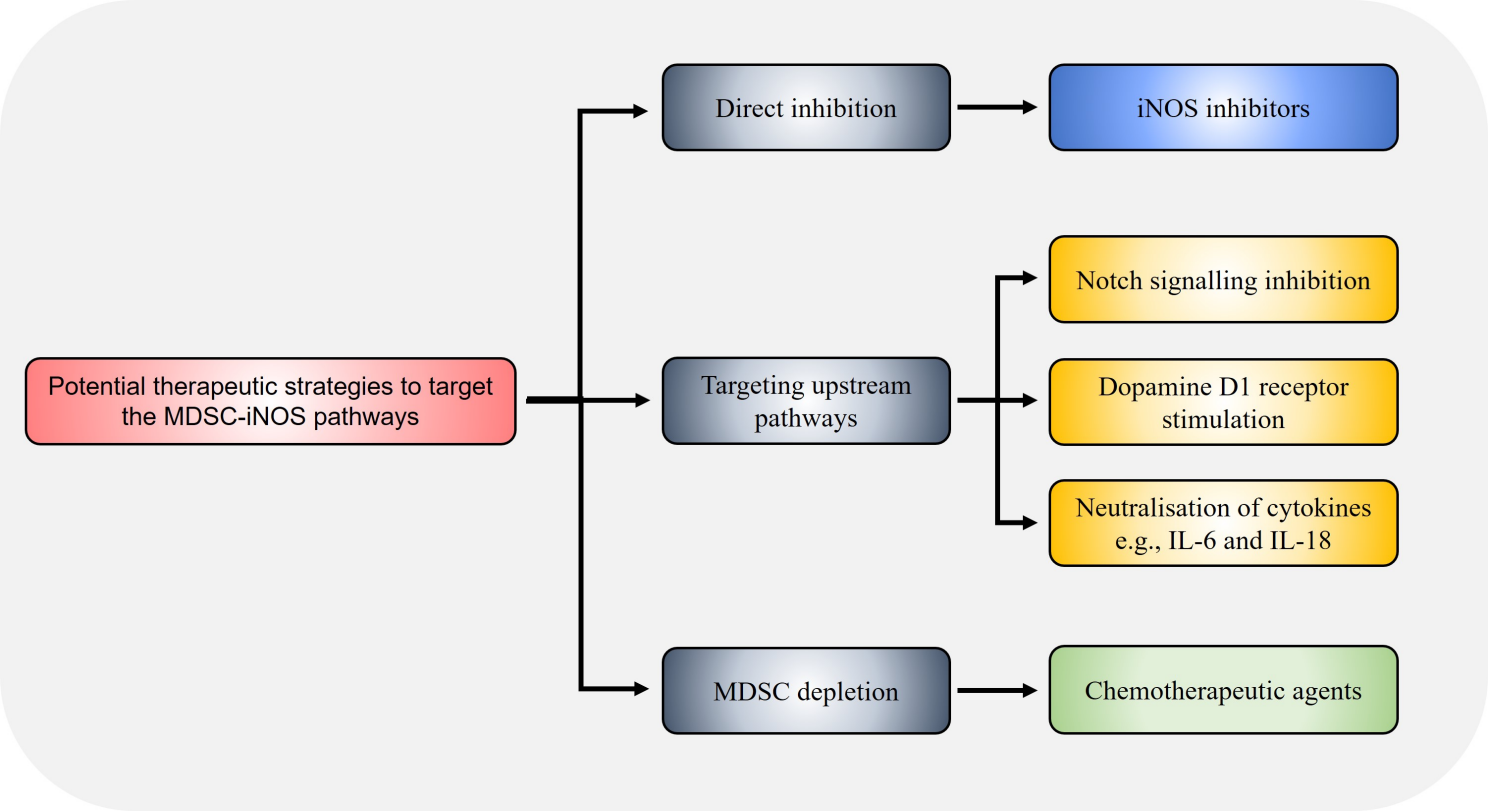
Potential strategies to target the MDSC-iNOS immunosuppressive pathway. Therapeutic targeting of the MDSC-iNOS pathway may be achieved through direct iNOS inhibition via specific inhibitors such as 1400w, or through dopamine signalling via D1-like receptors on MDSCs which suppresses iNOS expression. The chemotherapeutic agents docetaxel and doxorubicin have also been shown to reduce MDSC frequency and iNOS expression. Alternatively, pharmacological inhibition of upstream pathways, for example through CTX014 antibody-mediated blockade of the Notch ligands Jag1 and Jag2, or neutralisation of the cytokines IL-6 and IL-18, similarly reduce MDSC immunosuppressive capacity via decreasing iNOS expression

Strategies to decrease iNOS expression and resultant immunosuppressive activity in MDSCs may be achieved through targeting other IFNγ-independent upstream pathways that induce iNOS expression (Figure 3). PDE5 inhibition suppressed iNOS in MDSCs resulting in enhanced anti-tumour efficacy of cell-based immunotherapy treatments in HCC mice [40]. Notch signalling is associated with iNOS expression [63], and CTX014 antibody-mediated blockade of the Notch ligands Jagged 1 (Jag1) and Jag2 reduced MDSCs suppressive function via inhibiting iNOS and Arg1 expression [64]. Treatment with all-trans retinoic acid (ATRA) supplements reduced splenic M-MDSCs and G-MDSCs in HCC-bearing mice, along with inhibiting iNOS, Arg1, S100A8 and S100A9 expression in G-MDSCs in tumours, resulting in increased CD8^+^ T cell infiltration consistent with enhanced anti-tumour immune activity [65]. The alkaloid SNG similar reduces both Arg1 and iNOS expression in MDSCs allowing enhanced anti-tumour activity against murine lung cancer [41], and MDSC accumulation in lung cancer was found to be iNOS-dependent as shown using iNOS knockout mouse models where tumour MDSC numbers were impaired [66]. The dopamine signalling pathway has been shown to inhibit MDSC function and enhance anti-tumour immunity across different mouse cancer models [67]. Mechanistically, dopamine directly binds to D1-like receptors expressed on MDSCs and inhibits IFNγ-mediated iNOS production by both tumour bearing mice and human MDSCs *in vitro*, leading to enhanced T cell proliferation. This indicates that combination treatment of dopamine pathway agonists together with IFNγ may prevent the unwanted side effect of IFNγ-mediated iNOS expression in MDSCs while preserving the anti-tumoural function of IFNγ.

Like Arg1, chemotherapy can also reduce MDSC iNOS expression. Chemotherapeutic treatment via docetaxel reduces MDSC Arg1 and iNOS activity showing comparable efficacy to pharmacological inhibition of both Arg1 and iNOS, which in turn releases the inhibition on T cells allowing anti-tumour effector function [68]. These findings are replicated by independent groups using other chemotherapeutic agents such as doxorubicin which similarly reduced MDSC associated genes including as iNOS in breast cancer [45]. Therefore, these results suggest that the efficacy of certain chemotherapies used in the clinic currently may in part be associated with inhibiting MDSC immunosuppressive function.

Under certain contexts, iNOS have been shown to be the main mechanism of MDSC-mediated T cell suppression through comparing different inhibitors against iNOS, Arg1, IDO and TGF-β, for example in glioma [69]. This raises the possibility that selective inhibition of iNOS may produce desired therapeutic effects against certain cancer types. Accordingly, direct targeting iNOS via inhibitors such as 1400w reduced MDSC expansion in both primary tumour and metastatic sites in a mouse model of lung cancer, and strikingly potentiated the anti-tumour treatment efficacy of anti-PD-L1 immune checkpoint inhibition [70]. This is replicated in other studies where entinostat, a class I histone deacetylase inhibitor, similarly potentiated the effect of anti-PD-1 treatment by inhibiting the immunosuppressive functions of both M-MDSCs and G-MDSCs through reducing MDSC Arg1 and iNOS expression [71]. There is, therefore, an exciting prospect to combine strategies that target iNOS expression in MDSCs together with existing immunotherapies such as immune checkpoint inhibitors in cancer treatment to achieve enhanced anti-tumour effects.

Cytokines and chemokines play key roles in MDSC recruitment, activation, and suppressive function including iNOS expression. In human oesophageal cancer patients, MDSCs and iNOS expression are increased compared to healthy controls (the latter by three-fold), and positively correlated with IL-6 levels [33]. Accordingly, IL-6 has been shown to promote MDSC frequency and iNOS expression in liver and bladder cancer, enhancing their ability to suppress both CD4^+^ and CD8^+^ T cells [72, 73]. Others have found IL-18 to promote MDSC accumulation in HCC while toll-like receptor 2 (TLR2) suppresses this pathway, as genetic TLR2 deficiency resulted in increased iNOS^+^ MDSCs and T cell suppression [74]. TLR2 agonists such as Pam3CSK4 may represent a viable therapeutic strategy. Experimentally, Pam3CSK4 treatment inhibited IL-18 expression resulting in reduced iNOS positive MDSCs and subsequent regression of liver cancer. Strategies that target IL-18 and indeed IL-6, for example through antibody-mediated cytokine neutralisation, may similarly be efficacious in reducing tumour MDSC frequency and iNOS expression. In lung cancer models, the chemokine monocyte chemoattractant protein-1 (CCL2) positively correlates with MDSC frequency, and antibody-mediated blockade of CCL2 reduces both G-MDSCs and M-MDSCs in tumours reducing iNOS and Arg1 expression [75]. The inhibition of MDSC function resulted in increased CD4^+^ and CD8^+^ T cell infiltration, enhanced IFNγ production and prolonged mice survival. Intriguingly, the increased IFNγ expression in this scenario upon CCL2 blockade and decrease in MDSC iNOS expression did not result in reciprocal IFNγ upregulation, indicating that therapeutic strategies that inhibit MDSC iNOS-mediated suppression can effectively boost anti-tumour immunity without being self-limiting.

## MDSC-mediated immunosuppression via ROS

Another major mechanism utilised by MDSCs to mediate immunosuppressive functions against anti-tumour immunity is through ROS, although the relative importance of this pathway differs depending on the subset of MDSC. Accordingly, while M-MDSCs may preferentially utilise Arg1 and iNOS pathways to suppress T cells, ROS-mediated T cell suppression for example through hydrogen peroxide (H_2_O_2_) is prominently displayed by G-MDSCs [76]. In G-MDSCs, STAT3 and NADPH oxidase (NOX) functioning has been shown to be raised, consistent with their ability to produce high levels of ROS. Nevertheless, studies in human MDSCs found that both CD11b^+^CD15^+^HLA-DR^low/–^ G-MDSCs and CD11b^+^CD14^+^HLA-DR^low/–^ M-MDSCs can upregulate ROS production in response to tumour-conditioned-supernatant and suppressed T cell proliferation *in vitro* [77]. This indicates that G-MDSCs and M-MDSCs can utilise ROS pathways to inhibit anti-tumour immunity, although likely to different degrees.

ROS are a group of more than 20 identified small molecules that contain oxygen, and are short-lived with high chemical reactivity [78]. Mechanistically, ROS such as the superoxide anions, H_2_O_2_ and hydroxyl radicals can directly induce cell death through autophagy-induced cell death or upregulation of the Fas pathway leading to apoptosis of anti-tumoral T cells, and this results in an immunosuppressive tumour microenvironment [79, 80]. Furthermore, ROS plays indispensable roles on MDSCs themselves, acting in an autoparacrine manner to maintain MDSCs in an undifferentiated state thereby preserving their immunosuppressive capacity [76]. ROS also upregulates the expression of the transcription factors hypoxia-inducible factor 1α (HIF-1α) and nuclear factor erythroid 2-related factor (Nrf2) in MDSCs, both of which in turn enhances MDSC-mediated T cell suppression through downstream mechanisms. For example, HIF-1α induces PD-L1 expression on MDSCs, which can then bind to PD-1 on T cells and prevent T cell effector function [81]. On the other hand, genetic deficiency of Nrf2 resulted in reduced MDSC frequency and increased survival of tumour-bearing mice [82]. Therefore, MDSC-derived ROS can both promote MDSC maintenance and kill T cells thereby crafting an immunosuppressive tumour microenvironment.

Various strategies have been proposed and devised to target MDSC ROS production. Direct targeting ROS production or release by MDSCs through NOX inhibitors or ROS scavengers such as *N*-acetylcysteine respectively, greatly reduced MDSC function and restored CD8^+^ T cell proliferation [83]. In glioma, hypoxia induced the production of miR-10a and miR-21 containing exosomes by gliomas, which in turn promoted MDSC frequency and ROS production [84]. Genetic knockout of miR-10a and miR-21 in gliomas largely abrogated their ability to sustain MDSCs, indicating that therapeutic targeting of this pathway may inhibit the MDSC-ROS pathway. Given that ROS is required for MDSC survival as discussed above, methods that suppress ROS production by MDSCs may also lead to MDSC cell death in addition to reducing suppressive capacity. IPI-549-mediated inhibition of PI3Kγ downregulates ROS leading to MDSC apoptosis, leading to enhanced CD8^+^ T cell infiltration in mouse CRC [39]. Pancreatic adenocarcinoma up-regulated factor (PAUF), a soluble protein associated with pancreatic cancer progression, promotes pancreatic cancer patient-derived MDSC suppressive function via induction of Arg1, iNOS and ROS expression, and this can be reversed through antibody-mediated blockade of PAUF suggesting it as a viable cancer treatment target [85]. The aforementioned SNG has also been shown to reduce ROS expression by lung cancer MDSCs leading to apoptosis [41]. Other upstream pathways of MDSC ROS expression may include the opioid signalling pathway. Treatment with the endogenous opioid peptide methionine enkephalin (MENK) reduced CT26 CRC tumour growth *in vivo*, and *in vitro* incubation of MDSCs with MENK led to reduced glycolysis and ROS production, indicating that opioid pathways may have anti-tumoural properties [86]. Tumour cell-derived granulocyte macrophage-colony stimulating factor (GM-CSF) activates MDSCs via inducing STAT3-mediated fatty acid transport protein 2 (FATP2), and pharmacological blockade of the latter via lipofermata reduced ROS expression, MDSC immunosuppressive activity, and tumour burden [87]. Furthermore, combination therapy of lipofermata with anti-PD-L1 immunotherapy showed potentiation resulting in increased CD8^+^ T cell activation and IFNγ production.

Targeted therapies currently used in the clinic, for example the aurora A kinase inhibitor alisertib, may act in part through inhibiting MDSC ROS production [88]. In that study, alisertib treatment inhibited STAT3-mediated ROS production in MDSCs, leading to increased CD8^+^ and CD4^+^ T cell activation, infiltration, and breast cancer elimination. Furthermore, combination therapy of alisertib with immune checkpoint inhibition via anti-PD-L1 treatment showed potentiation against breast cancer, consistent with the notion of MDSCs contributing to immunotherapy resistance. Chemotherapeutic agents have also been shown to reduce MDSC ROS production. In addition to reducing Arg1 and iNOS expression in MDSCs, doxorubicin similarly suppresses ROS release by MDSCs in breast cancer leading to increased T cell infiltration [45]. Cyclooxygenase (COX) inhibitors such as aspirin have long been used in the treatment and prevention of cardiovascular diseases, and is known to protect from cancers such as CRC [89]. MDSCs co-localises with COX-2 expression in tumour models, and treatment with the COX-2 inhibitor celecoxib inhibits MDSC ROS expression and suppressive function against T cells [90].

MDSCs preferentially utilise glycolysis for rapid ATP generation in order to sustain their survival, expansion and immunosuppressive function [91]. Interestingly, inhibition of glycolysis via blocking pyruvate dehydrogenase (PDH) inhibited MDSCs and reduced ROS generation resulting in tumour elimination [92]. Mechanistically, the glycolysis metabolite phosphoenolpyruvate exerts potent antioxidant properties thereby limiting excessive ROS accumulation within MDSCs thus allowing survival [91]. However, given that glycolysis is an essential metabolic pathway in homeostasis, systemic inhibitors are unlikely to be of use in human patients clinically due to the risk of toxicity, and methods for targeted inhibition in MDSCs should be explored.

## Other novel strategies of therapeutically targeting MDSCs

As mentioned, the lack of specific markers on MDSCs make direct targeting difficult. Therefore, any attempts to reduce MDSCs in cancer patients before specific markers are identified should focus on targeting upstream pathways or downstream MDSC immunosuppressive function. In this section, in addition to the numerous methods already discussed above, further potential strategies to target MDSCs are explored.

Cytokine-based therapies have recently gained traction in recent clinical trials, mainly through exploiting the anti-tumoural efficacy of type 1 immune cytokines [54]. MDSCs express a myriad of cytokine receptors, and targeting these may allow modulation of MDSC function. IL-36 is a recently characterised cytokine found to exert anti-tumoural activities [93]. Combination treatment using IL-36 with the currently clinically-utilised immunotherapeutic anti-PD-L1 enhanced anti-tumour therapeutic efficacy against melanoma [94]. Mechanistically, IL-36 reduced tumour MDSC frequency while increasing anti-tumoural M1 macrophages, indicating that IL-36 may induce MDSC differentiation. Importantly, one of the key features of human MDSCs in cancer patients is the downregulation of major MHC-II, making them less prone to present tumour epitopes thus refraining from stimulating CD4^+^ T cells [95]. Strategies to induce MDSC differentiation into macrophages or dendritic cells which expresses MHC-II, are appealing as this not only reduces immunosuppressive MDSCs in the tumour microenvironment, but also increases immunostimulatory myeloid cells (e.g., M1 macrophages) with the potential to stimulate anti-tumoural T cells. The potential ability of IL-36 to promote MDSC differentiation into M1 macrophages is fascinating though further experiments are required to ascertain this. Others have found that the plant-derived benzophenanthridine alkaloid SNG promotes MDSC differentiation into macrophages and dendritic cells through inducing the nuclear factor kappa-B (NF-κB) pathway leading to reduced lung cancer burden [41]. As the IL-36 signalling pathway similarly involves NF-κB, this may explain the ability of IL-36 to induce MDSC differentiation [96]. The traditional Chinese medicine Jianpi Huayu Decoction has also been found to promote MDSC differentiation into macrophages and dendritic cells in mice with HCC, leading to enhanced CD4^+^ T cell proliferation and tumour regression [97]. ATRA can induce MDSCs to differentiate into mature myeloid cells, and combination treatment with an anti-PD-L1 immune checkpoint inhibitor enhanced anti-tumour efficacy against cervical cancer [98]. ATRA reduces both M-MDSCs and G-MDSCs in HCC mouse models and similarly exert anti-tumoural activities [65].

MDSCs are potent producers of the immunosuppressive cytokine IL-10 [6], which plays a key role in mediating MDSC suppression of anti-tumour immunity (Table 2). IL-10 acts via binding to its heterodimeric receptor IL-10 receptor 1 (IL-10R1) and IL-10R2 expressed by a myriad of cell types exerting broad effects [99], and induces the transcription and expression of anti-inflammatory genes via STAT3 [100]. Due to its potent ability to suppress anti-tumour immunity, IL-10 is associated with poor prognosis across a broad range of human cancers as shown by recent meta-analyses encompassing multiple solid and haematological cancer types [101]. In mice with MC38 CRC, antibody-mediated neutralisation of IL-10 impaired the immunosuppressive activity of MDSCs [102]. Tumour and peripheral blood MDSCs isolated from human CRC cancer patients potently inhibited T cell proliferation *ex vivo*, and pharmacological inhibition of IL-10 but not Arg1 of iNOS reversed the immunosuppressive effect of MDSCs in a human CRC study [103]. In another study, human gastric cancer patient tumour MDSCs suppressed T cells *ex vivo* via IL-10, resulting in a decrease in CD4^+^ T cell production of IL-2 and IFNγ consistent with impaired effector function [104]. In that study, the immunosuppressive effect and IL-10 release by MDSCs were driven by the hormone vasoactive intestinal peptide (VIP), suggesting that targeting VIP may be another viable strategy to limit MDSC IL-10 production and function. The tumour promoting roles of IL-10^+^ MDSCs are not limited to solid tumours, as IL-10-producing CD14^+^HLA-DR^–^ MDSCs have also been observed in patients with non-Hodgkin’s lymphoma and negatively correlated with anti-tumoural NK cell abundance. Altogether, strategies that reduce MDSC-derived IL-10, whether through inhibiting upstream pathways or directly neutralising IL-10, is likely to be beneficial in the treatment of a range of human cancers.

**Table 2:** A comprehensive summary of studies on targetting the MDSC-IL-10 pathway

**Study**	**Model**	**Cancer type**	**Strategy to suppress MDSC function**
Rossowska et al. [102]	Murine	CRC	Concomitant anti-IL-10 treatment enhanced efficacy of cyclophosphamide and bone marrow-derived dendritic cell transfer therapy via limiting MDSC suppressive functions allowing enhanced NK cell anti-tumour effector function.
Rossowska et al. [115]	Murine	CRC	Silencing of IL-10 expression through inoculation of tumours with shRNA-IL-10 lentivectors inhibited MDSCs and suppressed cancer growth via enhanced anti-tumour type 1 immune response.
Gupta et al. [116]	Murine	Breast cancer (triple negative)	Oral atovaquone treatment reduced tumour MDSCs and IL-10 in HCC1806, CI66 and 4T1 paclitaxel-resistant (4T1-PR) breast cancer, leading to a reduction in tumour growth by 45%, 70% and 42% respectively.
Hu et al. [117]	Murine	HCC	HCC tumour MSDC-derived IL-10 suppressed the ability of dendritic cells to produce IL-12 and stimulate T cells.
Shen et al. [118]	Murine	Pancreatic cancer	IL-10 protein trap treatment reduced immunosuppressive cell function including MDSCs, leading to activation of anti-tumour cells such as NK cells thereby suppressing tumour growth.
Li et al. [104]	Human	Gastric cancer	Human gastric cancer patient-derived tumour MDSCs suppressed CD4^+^ T cells *ex vivo* via IL-10 in response to VIP, leading to reduced IL-2 and IFNγ production and impaired anti-tumour immunity. Inhibiting VIP may represent a viable strategy in targeting the MDSC-IL-10 pathway.
Gneo et al. [103]	Human	CRC	Blockade of M-MDSC-derived IL-10, but not Arg1 or iNOS, released human CRC tumour M-MDSC-mediated suppression on T cells and restored T cell proliferation.
Sato et al. [119]	Human	Lymphoma (non-Hodgkin’s)	Human lymphoma patients with higher IL-10 producing MDSCs (but not Arg1 or iNOS) are associated with reduced anti-tumoural NK cells in blood.

shRNA: short hairpin RNA

Finally, utilisation of existing chemotherapeutic agents such as doxorubicin, 5-FU and paclitaxel/docetaxel, which has been shown to deplete MDSCs in addition to their direct anti-tumoural actions on cancer cells, remains highly promising and may potentiate other existing treatment modalities when used in combination and overcome therapeutic resistance. Indeed, studies have shown that combination therapies involving depletion of MDSCs via chemotherapy prior to other treatment modalities such as immunotherapy greatly improved outcome in experimental models [105, 106]. Concomitant treatment with doxorubicin significantly increased the efficacy of adoptive T cell transfer leading to reduced breast cancer burden in mouse models [105]. Furthermore, prior depletion of MDSCs with the standard carboplatin-paclitaxel regimen in patients with advanced cervical cancer enhanced the efficacy of the human papillomavirus type 16 synthetic long peptides (HPV16-SLPs) vaccine (which protects against the oncogenic virus HPV16), characterised by a strong and sustained HPV16-specific T cell response [106]. On the other hand, strategies that target MDSC Arg1 (Figure 2) and iNOS (Figure 3) pathways are actively being developed, and clinical trials to establish their safety and efficacy are required. Similarly, neutralisation of cytokines involved in MDSC activation such as IL-6 are actively being pursued in ongoing clinical trials [107].

Ultimately, the identification of markers specific to MDSCs remains crucial and will open avenues for direct MDSC targeting which will reduce off-target effects and minimise therapeutic toxicities. One such strategy could be through targeting the marker CD84, where it was shown that MDSCs with increased CD84 expression showed enhanced ROS production and T cell suppression capacity in breast cancer [108]. Nevertheless, as CD84 has also been found to be expressed by other immune cell types including macrophages, B cells and T cells [109, 110], and in the latter contributes to stimulating IFNγ release [111], strategies that target CD84 to deplete or inhibit MDSCs may lead to off-target effects and simultaneously impair anti-tumour T cell activity. Similarly, tumour-infiltrating MDSCs have also been found to express heightened levels of the IL-4 and IL-13 receptors [28], and while antibody-mediated blockade of the IL-4 and IL-13 pathways are promising and actively explored in ongoing clinical trials [54], these cytokines have pleiotropic effects and non-specific blockade may lead to unwanted side effects. Further studies in this avenue are required and the identification of a specific targetable marker unique to MDSCs remains to be discovered.

## Conclusions

MDSCs play critical roles in eliciting therapeutic resistance against all major modes of cancer treatment including surgery, chemotherapy, radiotherapy and immunotherapy through a combination of immunosuppressive mechanisms. Increasing understanding of MDSC immunosuppressive mechanisms have proved invaluable in opening avenues for the development of potential targeting strategies against MDSCs by limiting their downstream effector function. While currently difficult to achieve clinically, methods that promote MDSC differentiation or reprogramme MDSCs towards an anti-tumoural phenotype will likely yield the most benefits to patients through directly stimulating T cells. Alternatively, identifying a universal marker that is unique to MDSCs will allow specific targeting for example through antibody-mediated depletion, with low risk of therapeutic toxicity as MDSCs predominantly exist under pathological states as is the case in cancer patients. Novel therapeutics that target MDSCs can be used either alone or in combination with existing therapies to overcome treatment resistance, and will become the mainstay in future cancer immunotherapies.

## Abbreviations

5-FU: 5-flurouracil
Arg1: arginase 1
ATRA: all-trans retinoic acid
C/EBPβ: CCAAT/enhancer-binding protein β
CCL2: chemokine monocyte chemoattractant protein-1
CI: confidence interval
COX: cyclooxygenase
CRC: colorectal cancer
CTLA-4: cytotoxic T-lymphocyte antigen-4
EGFR: epidermal growth factor receptor
G-MDSCs: granulocytic myeloid-derived suppressor cells
HCC: hepatocellular carcinoma
HER2: human epidermal growth factor receptor 2
HLA-DR: human leukocyte antigen-DR isotype
HR: hazard ratio
IDO: indoleamine 2,3-dioxygenase
IFNγ: interferon γ
IL-10: interleukin 10
ILC1s: type 1 innate lymphoid cells
iNOS: inducible nitric oxide synthase
Jag1: Jagged 1
MDSCs: myeloid-derived suppressor cells
MHC-II: major histocompatibility complex class II
miR-107: microRNA-107
M-MDSCs: monocytic myeloid-derived suppressor cells
MyD88: myeloid differentiation factor 88
NK cells: natural killer cells
NO: nitric oxide
OS: overall survival
PD-1: programmed cell death protein 1
PDE5: phosphodiesterase 5
PD-L1: programmed cell death ligand 1
PI3K: phosphoinositide 3-kinase
ROS: reactive oxygen species
SNG: sanguinarine
STAT6: signal transducer and activator of transcription 6
TGF-β: transforming growth factor β
Th1: T helper 1
TLR2: toll-like receptor 2
TREM2: triggering receptor expressed on myeloid cells 2
VIP: vasoactive intestinal peptide

## Declarations

### Copyright

© The Author(s) 2024.
